# Treatment of Anaerobic Digester Liquids via Membrane Biofilm Reactors: Simultaneous Aerobic Methanotrophy and Nitrogen Removal

**DOI:** 10.3390/microorganisms12091841

**Published:** 2024-09-05

**Authors:** Egidio F. Tentori, Nan Wang, Caroline J. Devin, Ruth E. Richardson

**Affiliations:** 1 School of Civil and Environmental Engineering, Cornell University, Ithaca, NY 14853, USA; nw323@cornell.edu (N.W.); cjd245@cornell.edu (C.J.D.); rer26@cornell.edu (R.E.R.); 2 Gradient, One Beacon St., Boston, MA 02108, USA

**Keywords:** wastewater treatment, membrane biofilm bioreactors, methanotrophs, anammox, nitrogen removal, denitrification, methane, nitritation

## Abstract

Anaerobic digestion (AD) produces useful biogas and waste streams with high levels of dissolved methane (CH_4_) and ammonium (NH_4_^+^), among other nutrients. Membrane biofilm reactors (MBfRs), which support dissolved methane oxidation in the same reactor as simultaneous nitrification and denitrification (ME-SND), are a potential bubble-less treatment method. Here, we demonstrate ME-SND taking place in single-stage, AD digestate liquid-fed MBfRs, where oxygen (O_2_) and supplemental CH_4_ were delivered via pressurized membranes. The effects of two O_2_ pressures, leading to different O_2_ fluxes, on CH_4_ and N removal were examined. MBfRs achieved up to 98% and 67% CH_4_ and N removal efficiencies, respectively. The maximum N removal rates ranged from 57 to 94 mg N L^−1^ d^−1^, with higher overall rates observed in reactors with lower O_2_ pressures. The higher-O_2_-flux condition showed NO_2_^−^ as a partial nitrification endpoint, with a lower total N removal rate due to low N_2_ gas production compared to lower-O_2_-pressure reactors, which favored complete nitrification and denitrification. Membrane biofilm 16S rRNA amplicon sequencing showed an abundance of aerobic methanotrophs (especially *Methylobacter*, *Methylomonas,* and *Methylotenera*) and enrichment of nitrifiers (especially *Nitrosomonas* and *Nitrospira*) and anammox bacteria (especially Ca. *Annamoxoglobus* and Ca. *Brocadia*) in high-O_2_ and low-O_2_ reactors, respectively. Supplementation of the influent with nitrite supported evidence that anammox bacteria in the low-O_2_ condition were nitrite-limited. This work highlights coupling of aerobic methanotrophy and nitrogen removal in AD digestate-fed reactors, demonstrating the potential application of ME-SND in MBfRs for the treatment of AD’s residual liquids and wastewater. Sensor-based tuning of membrane O_2_ pressure holds promise for the optimization of bubble-less treatment of excess CH_4_ and NH_4_^+^ in wastewater.

## 1. Introduction

Anaerobic digestion (AD) is used in wastewater treatment to reduce solid waste from sludge and recover CH_4_-rich biogas. AD effluent streams contain high levels of ammonium (NH_4_^+^) and Chemical Oxygen Demand (COD), including dissolved CH_4_ [1,2,3]. Inputs of NH_4_^+^ to water bodies represent an important factor leading to eutrophication, and nitrous oxide (N_2_O)—produced as a byproduct during conventional wastewater treatment—and CH_4_ are potent greenhouse gases (GHGs) [4,5]. CH_4_ and N_2_O emissions from wastewater treatment represent a major source of global anthropogenic GHG emissions [4,6]. In conventional wastewater treatment, biogas recovered from AD is used for energy or flared to minimize atmospheric GHG emissions. Even with biogas capture practices, fugitive CH_4_ emissions from wastewater treatment still represent a source of GHG emissions [1,6,7].

The AD process’s residual liquids (AD digestate) must be dealt with prior to release into receiving waters (refer to Table S1 in the Supplementary Materials for a list of abbreviations and terminology used in this study). Various biochemical treatment strategies have been developed that couple the removal of organic carbon, including dissolved CH_4_, and fixed nitrogen compounds. Several configurations can achieve simultaneous removal of CH_4_ and ammonia; however, either temporal or spatial niche partitioning is critical for aerobic and anaerobic processes to occur simultaneously.

In conventional wastewater treatment, nitrogen removal processes generally occur under physical or temporal separation, as nitrification is a largely aerobic process and denitrification requires anaerobic or anoxic conditions [8]. Biological N removal via anammox (i.e., oxidation of NH_4_^+^ to N_2_ using NO_2_^−^ as the electron acceptor [9]) is also a viable treatment option for the removal of nitrogen from wastewater [2,8,10]. Methanotrophs, which rely on CH_4_ as a carbon and energy source, have garnered attention as a treatment option for the simultaneous removal of CH_4_ and nitrogen from wastewaters, due to their ability to link C and N cycles by using NO_2_^−^ and NO_3_^−^ as electron acceptors and/or nitrogen sources for biomass growth [1,11,12,13]. For high-NH_4_^+^ wastewaters with low initial NO_2_^−^ levels under appropriate O_2_ conditions, nitrogen removal treatment options that combine partial nitritation and anammox are possible. Optimal dissolved O_2_ conditions (typically <2 mg L^−1^) are required in this process to minimize complete nitrification and prevent inhibition of anammox activity [14]. To fully treat CH_4_- and NH_4_^+^-rich wastewaters, such as AD effluents, methanotrophs and N-cycling bacteria would need to grow in concert to simultaneously treat the nutrient streams.

Membrane biofilm reactors (MBfRs) provide the spatial stratification required for aerobic and anaerobic processes, while retaining slow-growing organisms more effectively compared to suspended growth systems [15,16]. Compared to open aeration processes in conventional wastewater treatment, membrane-based technologies have lower GHG emissions by avoiding the stripping of dissolved CH_4_, as well as lower N_2_O emissions, aeration costs, and biosolids production [2,7,8,15]. In addition, membrane-based technologies are better suited for the treatment of high-nitrogen anaerobic effluents, including AD liquids [2]. Simultaneous nitrification and denitrification (SND) has been demonstrated experimentally in aerobic MBfRs [17,18,19,20], aerobic rotating biological contactors [21], and sequencing batch reactors [22]. Due to the varied requirements and growth rates, organism out-selection is a potential concern, and previous single-stage reactor studies have generally relied on synthetic wastewater with optimal NH_4_^+^/NO_2_^−^ ratios in the influent feed over using actual wastewater. However, coexistence of NH_4_^+^- and NO_2_^−^-oxidizing bacteria, denitrifiers, and anammox bacteria is possible with appropriate operational strategies, such as fine tuning of reactor O_2_ [16,18,23,24].

Methanotrophs include aerobic and anaerobic classes from both bacterial and archaeal lineages, with anaerobes using electron acceptors including sulfate, nitrate, nitrite, humic acids, and metals [25]. Methane oxidation with simultaneous nitrification and denitrification (ME-SND) has been previously demonstrated in attached-growth methanotroph reactors, achieving CH_4_ and nitrogen removal rates of 21 and 8 mg L^−1^ d^−1^, respectively [26,27,28]. ME-SND is appealing, as it reportedly improves the denitrification process, potentially due to methanotrophs’ production of organic intermediates. CH_4_ is readily available in wastewater treatment facilities as a product from the AD process and dissolved in AD wastewaters, and it yields N_2_ and CO_2_ as final products [7,16,29,30,31,32,33,34,35,36]. Under optimal conditions, ME-SND could take place in single-stage reactors for wastewater with high NH_4_^+^/CH_4_ contents and low NO_2_^−^ concentrations; however, this would require the appropriate, narrow range of ecological niches for the reactions catalyzed by these distinct microbial groups to be active [16,37].

The aim of this study was to investigate ME-SND in single-stage aerobic MBfRs for the treatment of CH_4_- and NH_4_^+^-rich AD digestate from a full-scale wastewater treatment facility. The effects of inoculating reactors with methanotrophs, hydraulic retention time (HRT), and membrane O_2_ and CH_4_ pressures on reactor performance were explored, as were the effects of adding NO_2_^−^ during both batch and continuous operations. Additionally, the effects of these variables on shaping the biofilm microbial communities were determined using bulk biofilm thickness characterization and 16S rRNA gene sequencing.

## 2. Materials and Methods

### 2.1. Reactor Setup

**Figure 1:**
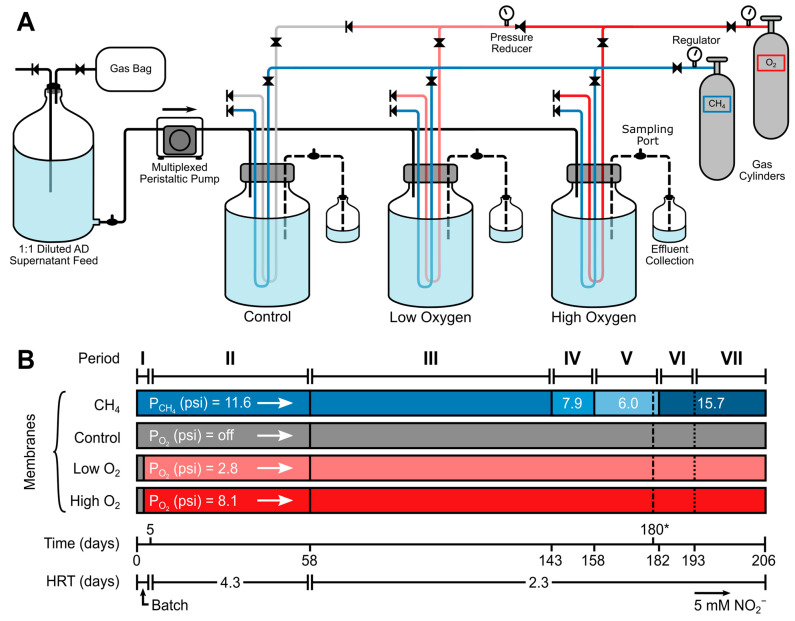
(**A**) Membrane biofilm reactor setup; (**B**) operational periods and membrane conditions. Operational periods are denoted in roman numerals (I–VII). Short-term NO_2_^−^ spike test date, day 180 (dashed line), indicated with an asterisk (*). On day 58, the flow rate increased, decreasing the HRT from 4.3 to 2.3 days. On day 193 (dotted line), the reactor feed tank was amended with 5 mM NO_2_^−^. O_2_ membrane pressures were turned on 2.5 days after startup, and the control reactor received no membrane O_2_. Pressures are denoted in psi (gauge).

Previously described hollow-fiber membrane bioreactors [38] with membranes for the delivery of gases and biofilm growth were used (Figure 1A); see Table S2 in the Supplementary Materials for membrane properties.

The reactors were operated as chemostats at room temperature (22 ± 3 °C), continuously stirred, with a 0.80 L liquid volume and 0.27 L headspace volume. The membranes were connected to either a CH_4_ (≥99.5% purity, Airgas, Radnor, PA, USA) or O_2_ (≥99.99% purity, Airgas) compressed gas cylinder. Pressures were set using regulators, verified regularly, and adjusted using an Omega PCL425 Pressure Calibrator as needed (Omega Engineering Inc., Norwalk, CT, USA). HRTs were controlled by a multiplexed peristaltic pump (Ismatec, Wertheim, Germany). The reactors were fed secondary anaerobic digester (AD) digestate (see Table S3 for the AD digestate’s chemical composition) from the Ithaca Area Wastewater Treatment Facility in Ithaca, NY, USA. AD digestate was collected every 4–7 days, stored in a gas-tight carboy at 4 °C, and used within a week. Prior to use, the AD digestate was diluted 1:1 with Milli-Q water to avoid peristaltic pump clogging. The AD feed tank was a gas-tight 20 L Pyrex glass carboy (Corning, Corning, NY, USA) connected to a CH_4_-filled 10 L gas bag (Zefon International, Inc., Ocala, FL, USA) to keep the dissolved CH_4_ levels elevated and minimize O_2_ infiltration.

### 2.2. Reactor Startup and Operating Conditions

The reactor startup conditions are summarized in Table 1. The experimental conditions tested included the addition of initial mixed methanotroph inoculation (see Supplementary Materials) and membrane O_2_ pressure.

The initial membrane pressures were chosen based on the CH_4_ and O_2_ molar ratios and expected gas permeation for the given membrane dimensions [28]. All reactors had an initial 5-day batch period (Period I) for membrane biofilm growth before continuous operation and with no additional O_2_ for 2.5 days (Figure 1B), in order to avoid O_2_ stress prior to setting the pressures of the high-O_2_ and low-O_2_ reactors to 8.1 and 2.8 psig, respectively. An in-line pressure regulator decreased the pressure for low-O_2_ reactors (Figure 1A). The reactors’ operational periods and additional operational changes are shown in Figure 1B and Table S5.

The reactors were operated continuously for 206 days, with average HRTs of 4.34 ± 0.11 (Period II) and 2.29 ± 0.05 days (Periods III–VI). In Periods IV, V, and VI, the membrane CH_4_ pressures were changed, leading to different CH_4_/O_2_ membrane loading ratios (Table S4). To explore the effects on total N removal, the AD feed was amended with 5 mM NO_2_^−^ in Period VII. On day 170 (Period V), the control reactor (R0) was knocked from its stir plate, causing significant membrane biofilm sloughing. Biomass samples were collected from the control reactor, and operations resumed.

### 2.3. Analytical Methods

Dissolved CH_4_, O_2_, and N_2_ concentrations were determined from headspace measurements using GC-TCD [38]; N_2_ was measured from day 83 (Period III) onwards. Due to atmospheric O_2_ and N_2_ interference, the minimum measurable dissolved concentrations corresponded to ~0.6 mg L^−1^. Nitrous oxide (N_2_O) reactor concentrations were determined from headspace samples on select days in Period III according to published GC methods [40]. Reactor liquid samples were collected every ~4.5 days and filtered using 0.22 μm syringe filters (Merck, Darmstadt, Germany) to determine dissolved PO_4_^3−^, NH_4_^+^, NO_2_^−^, and NO_3_^−^; PO_4_^3−^ was measured until day 152. Reactor pH and COD, using CHEMetrics COD Vials Kit K-7365 (CHEMetrics, Inc., Midland, VA, USA), were measured in unfiltered samples. NH_4_^+^ and PO_4_^3−^ were determined using previously published colorimetric methods [41,42], and NO_2_^−^ and NO_3_^−^ were determined using ion chromatography [40]. COD and NH_4_^+^ readings were performed on a Tecan Infinite M200 Pro microplate reader (Tecan US, Inc. Raleigh, NC, USA). On select days, suspended biomass was determined as total suspended solids (TSS) using standard methods [43]. The AD supernatants’ organic nitrogen and total alkalinity were determined using the Nitrogen s-TKN™ Vial Test Kit TNT880 (HACH Company, Loveland, CO, USA) and the titration method, respectively [44]. Reactor performance was evaluated by CH_4_ and NH_4_^+^, NO_2_^−^, and NO_3_^−^ (total inorganic nitrogen, N_Tot_) removal rates compared to the AD feed loading rate. CH_4_ and O_2_ consumption rates were determined using membrane pressures, measured dissolved gas levels, and a previously published permeation model [38].

### 2.4. Batch NO_2_^−^ Tests

Short-term NO_2_^−^ spike tests on day 180 (Period V, Figure 1B) were performed to observe reactor nitrogen cycling under batch conditions. During this time, the influent was stopped for ~24 h, and the CH_4_ and O_2_ pressures were kept constant. At time zero, NO_2_^−^ was added to all reactors to increase the dissolved concentrations in the reactors by 2 mM. Gas and liquid samples were collected every few hours as described above. Continuous operation resumed at the conclusion of the tests.

### 2.5. Biomass and Biofilm Sampling

Suspended biomass samples from the control reactor (day 170) and AD feed influent (days 40 and 140) were pelleted, frozen, and processed as previously described [38]. On day 206, the membrane assemblies were removed, and biofilm samples were collected from membrane segments using sterilized razor blades (Figure S6) to determine biofilm thickness, biomass, and average reactor solid retention times (SRTs), as well as for nucleic acid extraction and microbial community analysis (see Supplementary Materials).

### 2.6. Nucleic Acid Extraction, Sequencing, Assembly, and Microbial Community Analyses

Nucleic acid extraction and DNA quality checking followed previous methods [38], DNA was PCR-amplified with primer set 515F-806R, targeting the V4 region of the 16S rRNA gene [45], on a T100 Thermal Cycler (Bio-Rad, Hercules, CA, USA) using Q5^®^ Hot Start High-Fidelity 2X Master Mix (New England Biolabs, Ipswich, MA, USA). Gene amplicons were submitted to the Cornell Biotechnology Resource Center (BRC) Genomics Facility for quality control, library preparation, and sequencing. Sequencing was performed using the Illumina MiSeq platform with 2 × 250 paired-end read lengths. Raw sequences were demultiplexed and analyzed using the QIIME 2 (https://qiime2.org/ (accessed on 17 April 2021)) pipeline [46]. Reads were denoised and clustered into amplicon sequence variants (ASVs) using DADA2 v1.20 [47], with a max EE value of 6, and ASVs were annotated for taxonomy against the SILVA 138 99% database (https://arb-silva.de (accessed on 17 April 2021)) [48]. Data analysis and visualization were performed with the R package *Phyloseq* v1.38.0 [49], while Bray–Curtis dissimilarities for Principal Coordinate Analysis (PCoA) and diversity indices were determined using the R package *vegan* v2.5-7. Sequencing data were submitted to the NCBI database under submission: SUB12631397; BioProject ID: PRJNA928688.

## 3. Results

### 3.1. General Reactor Performance

**Figure 2:**
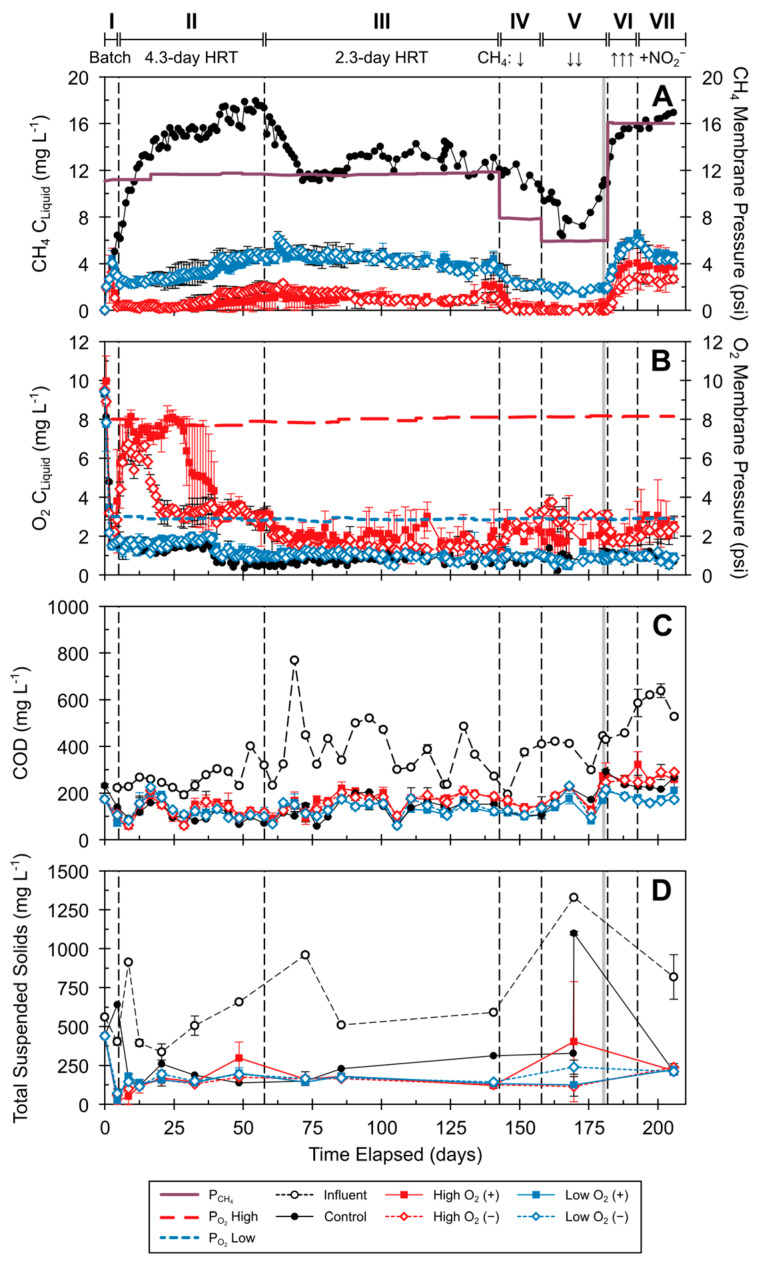
MBfRs’ dissolved CH_4_, O_2_, COD, and TSS: (**A**) dissolved CH_4_ and membrane pressure; (**B**) dissolved O_2_ and membrane pressure; (**C**) COD; (**D**) TSS. Operational periods are denoted in roman numerals (I–VII). Upward (↑) and downward (↓) arrows indicate an increase or decrease in CH_4_ pressure, respectively. Vertical dashed black lines: operational periods; gray lines: batch period with NO_2_^−^ addition. Error bars represent standard deviations from duplicate reactor measurements for each condition. See Figure 1 and Table 1 and Table S3 for details on operational periods.

The MBfRs were operated for a total of 206 days at two different HRTs and four CH_4_ pressures, for total of seven distinct operational periods (Figure 1). The effects of HRTs, O_2_, CH_4_/O_2_ loading ratios, and the addition of NO_2_^−^ to the influent feed on CH_4_ and fixed N removals were explored (Figure 2).

The dissolved CH_4_ and O_2_ concentrations in the reactors depended on the CH_4_ and O_2_ membrane pressures and microbial transformation. Periods I–III (CH_4_ pressures ~11.6 psig) had generally consistent dissolved CH_4_ concentrations throughout operation, with the averages stabilizing during Period III (2-day HRT) at 13.3, 1.0, and 3.8 mg L^−1^ for the control (R0), high-O_2_ (R1–R4), and low-O_2_ (R5–R8) reactors, respectively. Changes in CH_4_ pressure in Periods IV–VII affected the dissolved CH_4_ concentrations in all reactors. Halving the CH_4_ pressure to 5.9 psig in Period V decreased the average dissolved CH_4_ concentrations to 9.2, 0.1, and 1.9 mg L^−1^ for the control, high-O_2_, and low-O_2_ reactors, respectively. Increasing the CH_4_ pressure to 16 psig (Periods VI and VII) was accompanied by an increase in average dissolved CH_4_ concentrations for all conditions above the averages for Periods I–III. Lower dissolved CH_4_ concentrations were observed in the high-O_2_ reactors throughout operation. Overall, the experimental reactors were effective in transforming incoming CH_4_—both dissolved in the influent and from membrane permeation. Average O_2_ pressures of 8.0 ± 0.2 psig and 2.9 + 0.1 psig were maintained for the high-O_2_ and low-O_2_ reactors, respectively (Figure 2B), and the O_2_ levels stabilized by about day 40 for the remainder of Period II (4-day HRT) in all reactors, with values of 0.8, 1.2, and 3.7 mg/L for the control, low-O_2_, and high-O_2_ reactors, respectively. In Period III (2-day HRT), the average dissolved O_2_ concentrations were 0.9, 1.0, and 2.5 mg L^−1^ for the control, low-O_2_, and high-O_2_ reactors, respectively. Inoculation only affected the O_2_ levels in the early part of Period II. Inoculated high-O_2_ reactors had higher dissolved O_2_ concentrations compared to uninoculated high-O_2_ reactors, and similar concentrations were observed starting on day 50 (Figure 2B). Minimal transformation of CH_4_ was observed in the AD influent feed (Figure S2). Inoculation did not influence CH_4_ removal (open versus closed symbols in Figure 2A).

The reactors’ COD levels ranged between 100 and 200 mg COD L^−1^ across all conditions and varied with influent COD (Figure 2C). During Period II, the reactors had COD removal rates > 318 mg COD L^−1^ d^−1^ and efficiencies > 0.86 (Figure S3). In Period III, with decreased HRT and increased COD loading rate, the removal efficiencies decreased (average efficiencies ranging from 0.70 to 0.77); however, the COD removal rates were comparable to those in Period II (294 mg COD L^−1^ d^−1^; Figure S3). COD removal was impacted by influent feed COD and supplemental CH_4_ membrane permeation, as observed during Periods VI and V (low CH_4_ pressure) and Periods VI and VII (high CH_4_ pressure) (Figure 2C).

The reactors’ TSS did not differ significantly across reactor conditions (Figure 2D); the drastic increase in the control reactor’s TSS in Period V was due to sloughing on day 170. The average SRT (Table S6) of all reactors was 71.1 days (reactor averages of 62.4–78.0 days) during Period II and 40.5 days (reactor averages of 12.1–56.8 days) for Periods III–VII. The reactor pH levels were generally between 6 and 8 for all reactors, with a short-period pH below 6 in high-O_2_ reactors (Figure S1B). The control reactor’s pH was most similar to the influent pH, and slightly lower pH was observed in the experimental reactors (Figure S1B). In the experimental reactors, longer HRTs (Period II) resulted in lower reactor pH compared to shorter HRTs (Periods III–VII).

### 3.2. Fixed Nitrogen Transformations and Removal

**Figure 3:**
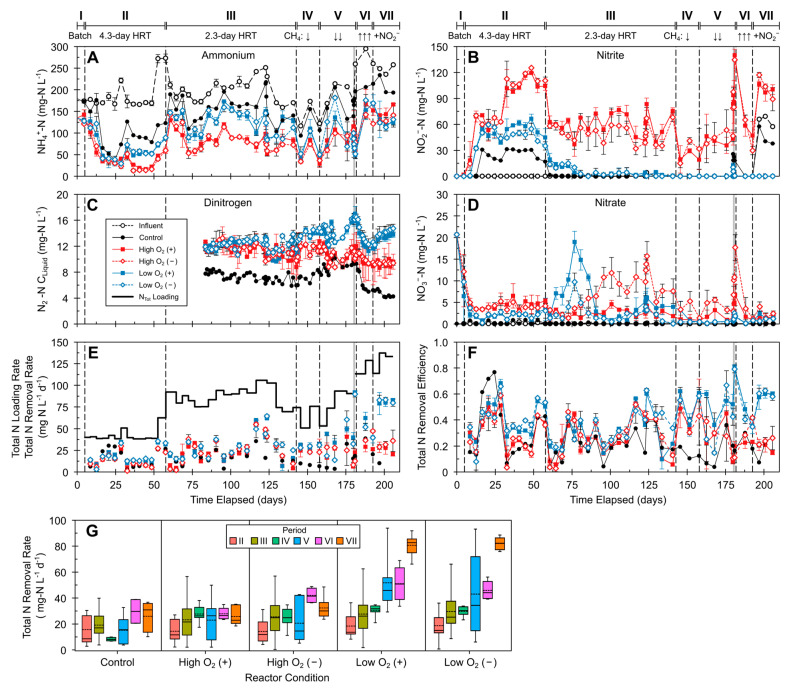
Dissolved nitrogen species and nitrogen removal performance of AD supernatant-fed MBfRs: (**A**) NH_4_^+^-N; (**B**) NO_2_^−^-N; (**C**) dissolved N_2_-N; (**D**) NO_3_^−^-N; (**E**) total inorganic nitrogen (N_Tot_ = NH_4_^+^-N + NO_2_^−^-N + NO_3_^−^-N) influent loading and removal rates; (**F**) N_Tot_ removal efficiency; (**G**) N_Tot_ removal rates by period. Operational periods are denoted in roman numerals (I–VII). Upward (↑) and downward (↓) arrows indicate an increase or decrease in CH_4_ pressure, respectively. For (**A**,**B**), vertical dashed black lines = operational periods; gray lines = batch activity periods; error bars = standard deviations from duplicate reactor measurements. For (**G**), error bars = 95% confidence intervals; medians = solid lines; means = dashed lines. See Figure 1 and Table 1 and Table S3 for details on operational periods.

Key nitrogen species data are shown in Figure 3. The influent nitrogen consisted almost entirely of NH_4_^+^, with concentrations between ~125 and 275 mg NH_4_^+^-N L^−1^ due to variations in the AD supernatant, while the influent NO_2_^−^ and NO_3_^−^ levels were below the 0.07 mg N L^−1^ detection limit (Figure 3A,B). Organic-bound nitrogen was also present in the AD influent and was typically about half the N concentration of NH_4_^+^-N (Table S3).

Initial inoculation did not affect the overall reactor nitrogen removal, and O_2_ pressure had the biggest effect on nitrogen transformation.

The reactors achieved their lowest effluent NH_4_^+^ concentration in Period II, accompanied by an increase in NO_2_^−^ concentration, reaching maxima of about 30, 120, and 60 mg NO_2_^−^-N L^−1^ for control, high-O_2_, and low-O_2_ reactors, respectively. Lowering the HRT in Period III to 2.3 days led to lower NO_2_^−^ levels in all reactors compared to Period II, with average concentrations of 55.3 and 4.4 NO_2_^−^-N L^−1^ for high-O_2_ and low-O_2_ reactors, respectively. In Periods IV and V, the decreased CH_4_ pressure was accompanied by a decrease in NO_2_^−^ concentrations in high-O_2_ reactors, while CH_4_ pressure changes did not affect the NO_2_^−^ levels in low-O_2_ reactors, which had levels consistently < 0.1 NO_2_^−^-N L^−1^.

In Periods III–V (2.3-day HRT), the reactors’ NH_4_^+^ concentrations were largely dependent on influent NH_4_^+^, with reactor concentrations around 75 and 125 mg NH_4_^+^-N L^−1^ for high-O_2_ and low-O_2_ reactors, respectively, while the control reactor NH_4_^+^ levels were similar to the influent levels. The decrease in CH_4_ pressure in Period V caused low-O_2_ reactors’ NH_4_^+^ levels to increase, while those of high-O_2_ reactors remained unchanged. Following the addition of 70 mg NO_2_^−^-N L^−1^ (5 mM NO_2_^−^) to the influent in Period VII, low-O_2_ reactors consistently achieved lower NH_4_^+^ concentrations compared to high-O_2_ reactors. Influent NO_2_^−^ in Period VII was not transformed in high-O_2_ and control reactors, while the low-O_2_ reactors’ NO_2_^−^ levels remained <0.1 NO_2_^−^-N L^−1^, indicating rapid transformation of both exogenous and nitrifier-produced NO_2_^−^.

Other than temporary increases in NO_3_^−^ levels in low-O_2_ reactors (days 70–90) and uninoculated high-O_2_ reactors (days 95–122), the NO_3_^−^ concentrations were ≤5 mg NO_3_^−^-N L^−1^ for the experimental reactors and below the detection limit in the control reactor (Figure 3D). High ratios of NO_2_^−^ to NO_3_^−^ levels in high-O_2_ reactors indicated a preference for partial nitrification over complete nitrification. The N_2_ concentrations were higher in experimental reactors compared to both the control reactor and the AD influent (Figure 3C and Figure S2). In Periods IV–VII, the low-O_2_ reactors’ N_2_ concentrations were consistently ~2 mg N_2_-N L^−1^ higher compared to high-O_2_ reactors, reaching a maximum difference in Period VII of 4.3 mg N_2_-N L^−1^, coinciding with the addition of NO_2_^−^ to the influent.

### 3.3. Total Inorganic Nitrogen (N_Tot_) Removal

The total inorganic nitrogen influent loading and removal rates of the membrane bioreactors are shown in Figure 3E. During Periods I–VI, the N_Tot_ influent loading consisted almost entirely of NH_4_^+^; additional NO_2_^−^ was added to influent in Period VII. In Period II, with comparable NH_4_^+^ removal in all reactors, the low-O_2_ and control reactors had better N_Tot_ removal rates, as less NO_2_^−^ and NO_3_^−^ were produced compared to high-O_2_ reactors. The maximum N_Tot_ removal rate by low-O_2_ reactors during Period II was 36.2 mg N L^−1^ d^−1^, with removal efficiencies ranging between 0.6 and 0.8 (Figure 3E,F). After decreasing the HRT in Period III, the N_Tot_ loading rate increased, and the experimental reactors’ removal rates generally remained between 25 and 75 mg N L^−1^ d^−1^ (removal efficiencies of 0.2–0.6) until the end of Period IV. The control reactor’s removal rate and efficiency steadily decreased with continued operation. Greater NH_4_^+^ conversion and NO_2_^−^ and NO_3_^−^ accumulation, and therefore lower N_Tot_ removal rates, in high-O_2_ compared to low-O_2_ reactors continued throughout the reactors’ operation. The addition of NO_2_^−^ (Period VII) led to the biggest difference in nitrogen removal rates and efficiencies between the reactor O_2_ conditions. High-O_2_ reactors’ performance was not affected, while low-O_2_ reactors had a maximum N_Tot_ removal rate of 91.7 mg N L^−1^ d^−1^, among the highest during this study. N_Tot_ loading and CH_4_ pressure did not strongly affect the control and high-O_2_ reactors’ N_Tot_ removal rates, while the low-O_2_ reactors’ performance steadily increased throughout operation (Figure 3G). The addition of NO_2_^−^ to the influent had the biggest impact on nitrogen removal in low-O_2_ reactors. The N removal rates for the reactors are summarized by period in Table S7.

N_2_O emissions from nitrification and denitrification treatment of wastewater can be significant [50] and are strongly associated with influent O_2_ levels and NH_4_^+^-N loading [51]. Reactor N_2_O amounts were measured on three selected sampling dates in Period III (Figure S4); N_2_O production was only observed in experimental reactors where O_2_ was provided. Inoculation showed no effect on N_2_O levels in high-O_2_ reactors, with N_2_O levels ranging between 1.10 and 1.45 mM. The N_2_O levels in low-O_2_ reactors varied considerably; inoculated and uninoculated low-O_2_ reactors had N_2_O concentrations of 1.72–2.33 and 0.001–0.624 mM N_2_O, respectively.

### 3.4. Short-Term NO_2_^−^ Addition Tests

The 24-hour NO_2_^−^ spike tests (batch tests with 2 mM NO_2_^−^ added) were conducted on day 180 to observe reactor nitrogen transformation and determine potential NO_2_^−^ limitation and anammox potential (Period V; Figure S5). No significant changes were observed in dissolved gases (O_2_, CH_4_, and N_2_), while high-O_2_ reactors had higher NO_2_^−^ and NO_3_^−^ production compared to low-O_2_ reactors. NO_2_^−^ disappearance and NO_3_^−^ production in low-O_2_ reactors were potential indicators of denitrification and anammox.

### 3.5. Membrane Biofilm Characteristics

Biofilm growth was visible on all reactor membranes after Period I. The biofilms were uniform in color but varied in thickness, likely influenced by the reactor mixing conditions (Figures S6–S9; further discussion in the Supplementary Materials). O_2_ membranes (60 cm) accounted for a higher fraction of overall biomass compared with CH_4_ membranes (20 cm). The fraction of biofilm biomass in the CH_4_ membrane was higher in high-O_2_ reactors compared to low-O_2_ reactors (Figure S6A). Total biofilm biomass > 1250 mg was observed in all experimental reactors (Figure S6B). While the CH_4_ membrane biofilms in high-O_2_ reactors were generally thicker compared to low-O_2_ reactors, the O_2_ membrane biofilm thicknesses were similar across all reactors (Figure S6C,D).

### 3.6. Biofilm Microbial Community Structure and Diversity

**Figure 4:**
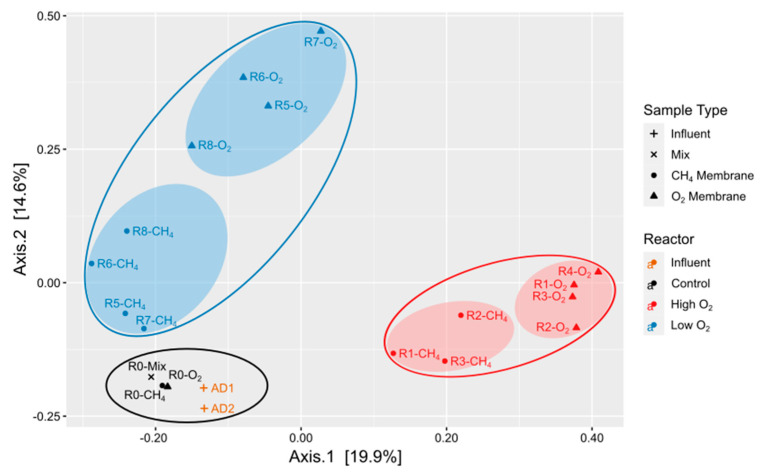
PCoA showing membrane biofilm microbial community samples. Bray–Curtis dissimilarity measurements. Circles denote sample clustering. R4-CH_4_ not included due to low number of reads.

Clustering by reactor O_2_ condition (Figure 4), consisting of AD influent and control, low-O_2_, and high-O_2_ reactors, was observed in the PCoA, and high-O_2_ and low-O_2_ samples were distinguished along Axis 1 and Axis 2, respectively. The community in the control reactor was most similar to that of the AD feed samples, whereas inoculation showed no impact on the experimental reactors’ microbial diversity by day 206.

**Figure 5:**
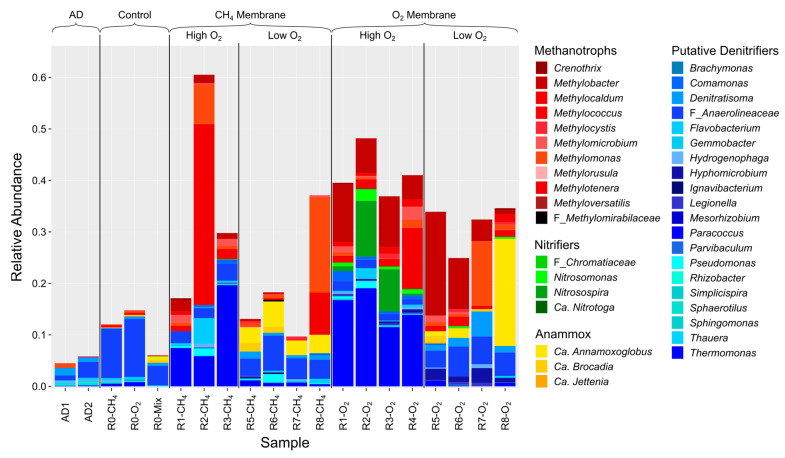
Genus-level taxonomic composition of AD and membrane biofilm samples of genera involved in CH_4_ and N cycling. R4-CH_4_ not included due to low number of reads.

Additional clustering by membrane was observed for high-O_2_ and low-O_2_ reactors, where the O_2_ membrane microbial communities were most distinct from both the AD feed and the control reactor. The AD feed and control reactor exhibited the highest Fisher diversity, followed by high-O_2_ reactors and low-O_2_ reactors with the lowest Fisher diversity (Figure S10). Differences in the Fisher diversity of the reactor biofilm microbial communities compared with the AD supernatant and due to O_2_ conditions for the experimental reactors were statistically significant (Kruskal–Wallis test, *p* < 0.005, Figure S10). Genus-level taxonomic classifications of ASVs from 16S rRNA membrane biofilm sequencing samples of genera involved in CH_4_ and fixed nitrogen metabolism are summarized in Figure 5.

Relevant microbial groups highlighted in Figure 5 include methane-oxidizing bacteria (MOB), nitrifiers (including ammonium-oxidizing bacteria (AOB) and nitrite-oxidizing bacteria (NOB)), putative denitrifying bacteria (DNB), and anammox bacteria. The control reactor samples most resembled the AD samples in both overall community composition and abundance of CH_4_- and N-cycling microorganisms. The R0-Mix sample (day-170 sloughed biofilm) had >1% anammox organisms (*Ca. Anammoxoglobus* and *Ca. Brocadia)*, higher than the day-206 membrane biofilms; these organisms were likely reestablishing on the R0-O_2_ membrane (<1% of the population).

Enrichment of CH_4_- and N-cycling microbes was observed in the experimental reactors compared to the AD feed and control samples (summed relative abundances of 4.5–14.8% and 9.7–60.6% for the experimental reactors and AD feed/control reactor, respectively). For both reactor conditions, aerobic MOB (*Methylobacter*, *Methylomonas*, and *Methylotenera*) were present at high relative abundances in both membranes in high-O_2_ reactors, while O_2_ membranes were preferred in low-O_2_ reactors. Methanotroph inoculum organisms were present at significantly lower abundances compared to other MOB, and long-term effects on microbial community due to inoculation were not observed. NC10 phylum anaerobic MOB (family *Methylomirabilaceae*) were measured in R6-CH_4_ samples, with relative abundance < 0.5%.

Nitrifiers (genera *Nitrosomonas*, *Nitrosospira*, and *Ca. Nitrotoga*) preferred O_2_ over CH_4_ membranes in high-O_2_ reactors, with lower abundances in low-O_2_ reactors. Putative denitrifiers (genera *Comamonas*, *Flavobacterium*, *Denitratisoma*, and *Thermomonas*) were found at similar relative abundances across all reactor samples; the genus *Hyphomicrobium* was observed in O_2_ membranes in low-O_2_ reactors and was absent in high-O_2_ reactors. Organisms from the *Anaerolineaceae* family, mainly consisting of anaerobic fermenters commonly found in wastewater [52,53], were found in all reactors, with higher relative abundances in low-O_2_ and control reactors compared to high-O_2_ reactors. The family *Anaerolineaceae* includes potential denitrifiers [54] often found alongside anammox organisms [55] and previously reported in both anammox and nitrite-dependent denitrifying anaerobic methane oxidizer (DAMO) reactors [24,56]. Anammox bacteria (family *Brocadiacea*) were absent in high-O_2_ reactors, while they represented a significant portion of reads in CH_4_ and O_2_ membrane biofilms (from ~1% up to 20.9% of reads) in low-O_2_ reactors. The majority of anammox bacteria were from the genus *Ca. Anammoxoglobus*, followed by the genus *Ca. Brocadia*.

## 4. Discussion

MBfRs performed simultaneous removal of CH_4_ and N from CH_4_- and NH_4_^+^-rich anaerobic digester effluent. Low-O_2_ conditions yielded better total fixed N removal compared to high-O_2_ conditions, as lower O_2_ levels allowed for partial nitritation coupled with anammox. High-O_2_ conditions yielded higher oxidation of NH_4_^+^ to NO_2_^−^ but decreased transformation to NO_3_^−^. Low-O_2_ reactors achieved nitrogen and CH_4_ removal efficiencies of up to 67% and >97%, respectively. High COD removal efficiencies (generally 80–90%) were observed for both sets of experimental reactors throughout operation. The CH_4_ and N removal efficiencies in this study were comparable to those of a two-stage anoxic–oxic membrane bioreactor system for the treatment of upflow anaerobic sludge blanket (UASB) reactor effluent (60% N removal and 95% CH_4_ removal) [57]. The methanotroph populations observed by Sánchez et al. consisted of phylum NC10 anaerobic methanotrophs and aerobic methanotrophs [57], while the methanotroph population in this study consisted almost entirely of proteobacterial aerobic methanotrophs. Similar to this study, high abundances of proteobacterial methanotrophs (genera *Methylococcus* and *Methylocystis*) and heterotrophic denitrifiers were observed in MBfRs capable of nitrite reduction [35].

The reactors in this study (maximum removal rates of 57–94 mg N L^−1^ d^−1^) achieved higher nitrogen removal rates compared to nitrite-dependent DAMO membrane bioreactors (20–40 mg N L^−1^ d^−1^) [17,58,59]. Despite having a lower membrane surface area/working volume ratio, the maximum N removal rates in this study were comparable to those of membrane nitrite-dependent DAMO and anammox bioreactors, with rates ranging from 67.8 to 190 mg N L^−1^ d^−1^ [60,61,62]. Cao et al. demonstrated ME-SND in MBfRs capable of NH_4_^+^ removal rates of 38.09 mg N L^−1^ d^−1^, where the addition of CH_4_ promoted NH_4_^+^ removal and N_2_O production following denitrification [13]. ME-SND in MBfRs provided synthetic wastewater and O_2_/CH_4_ ratios of 1.47 and 2.1, comparable to the ratios in this study (Table S4), achieving NH_4_^+^ removal rates of 77.5 and 95 mg L^−1^, respectively [63]. Maximum N removal rates as high as ~1000 mg N L^−1^ d^−1^ have been reported for membrane biofilm nitrite-dependent DAMO/anammox systems [56,64]. However, these were for reactors inoculated with anammox/nitrite-dependent DAMO enrichment cultures, with >400-day operational periods, and provided with a synthetic influent containing both NH_4_^+^ and NO_2_^−^. Additionally, they had approximately 8-fold higher membrane surface-area-to-volume ratios compared to this study, and increasing the membrane surface areas for biofilm growth has been shown to achieve better N removal rates and efficiencies [61]. Higher membrane surface-to-volume ratios could improve the N removal rates observed in this study.

Elevated NO_2_^−^ and NH_4_^+^ concentrations can be inhibitory to relevant microbial groups, including AOBs, methanotrophs, and anammox bacteria. NO_2_^−^ levels in the 5 mM (70 mg N L^−1^) range have been shown to cause a ~50% decrease in activity in obligate NH_4_^+^ oxidizers [65]. NO_2_^−^ effects on NH_4_^+^-oxidizing activity seem to be organism-specific, with some organisms (*Nitrosomonas europaea* and *Nitrosospira multiformis*) tolerant of up to 20 mM (280 mg N L^−1^) with no effect on activity [66]. The NO_2_^−^ levels observed in this study were generally <5 mM (70 mg N L^−1^) throughout operation, while levels above 8 mM NO_2_^−^ were observed for high-O_2_ reactors in Periods II and VI (Figure 2). For anammox bacteria, reported inhibitory NO_2_^−^ concentrations range from 100 to 280 mg NO_2_^−^-N L^−1^ (7.1–20 mM NO_2_^−^) [67,68], depending on growth conditions, and inhibition effects are mostly reversible [69]. Anammox-inhibitory NO_2_^−^ levels are higher than the levels observed in this study, except for high-O_2_ reactors during Period II, batch tests, and Period VII (Figure 2D and Figure S5). The combination of high NO_2_^−^ and O_2_ levels and low pH observed before day 45 in high-O_2_ reactors could have affected the initial growth of anammox bacteria, leading to other organisms colonizing the membranes (e.g., nitrifiers). NH_4_^+^ levels up to 1000 mg NH_4_^+^-N L^−1^ are not inhibitory for anammox bacteria [67], and the NH_4_^+^ levels in this study were below this threshold. O_2_ levels can also reversibly inhibit anammox activity. Reported dissolved O_2_ levels for anammox inhibition vary by organism, ranging from microaerobic levels (<0.04–0.12 mg O_2_ L^−1^) to ~2 mg O_2_ L^−1^ [70,71,72,73]. No significant anammox growth was observed for high-O_2_ reactors, likely due to the O_2_ permeation rates resulting in higher dissolved O_2_ concentrations in the bulk liquid (≥2 mg O_2_ L^−1^). In low-O_2_ reactors, the bulk liquid O_2_ levels were near the method detection limit of 0.6 mg O_2_ L^−1^, and anammox bacteria were well represented in the O_2_ membrane biofilms despite their sensitivity to O_2_. This could be due to the anammox bacteria in low-O_2_ reactors being NO_2_-limited, as shown by the fast NO_2_^−^ transformation during batch tests (Figure S5D). Localized higher NO_2_^−^ production at the O_2_ membrane interface likely favored anammox growth within the O_2_ membrane biofilm. Growth and activity of microbial groups under otherwise-inhibitory O_2_ levels have been observed for attached-growth denitrifier, nitrite-dependent DAMO, and anammox reactor systems [18,21,74]. Therefore, the growth mode (e.g., suspended vs. attached) can lead to microbial activity even under bulk liquid substrate levels that can cause inhibition.

The CH_4_ removal efficiencies observed in this work were comparable to those of anammox/DAMO reactors, which typically have efficiencies ranging from 85 to 96% [64,75]. Modeling efforts have also demonstrated the feasibility of O_2_-permeation MBfRs for the simultaneous removal of NH_4_^+^ and CH_4_ in single-stage reactors. Chen et al. found high potential removal efficiencies of both CH_4_ and nitrogen via stratified microbial biofilms containing AOB, methane-oxidizing bacteria (MOB), anammox bacteria, and DAMO organisms [37,76]. Key challenges for the removal of nitrogen and dissolved CH_4_ in these reactors were HRT and O_2_ surface loading rate. As mentioned previously, control of O_2_ in these reactors is critical, as O_2_ is necessary for the oxidation of CH_4_ and partial oxidation of NH_4_^+^ to NO_2_^−^ but can inhibit the activity of DAMO and anammox microbes [67].

Methanotrophs have diverse nitrogen metabolisms, capable of using NH_4_^+^ and NO_3_^−^ as N sources for growth [77]. Alternatively, both NH_4_^+^ and NO_2_^−^ can inhibit methanotrophic activity, with effects highly dependent on their relative amounts, e.g., high NH_4_^+^ concentrations are more inhibitory at low CH_4_ concentrations compared to high CH_4_ concentrations, which is consistent with competitive inhibition at the enzyme’s active site [77,78]. NH_4_^+^ and NO_2_^−^ inhibition effects on methanotrophs generally vary by organism [78]. Given the activity and relative aerobic abundances of aerobic methanotrophs in the MBfRs, inhibition due to NH_4_^+^ and NO_2_^−^ was assumed to be insignificant.

Due to the similarity of methane monooxygenase (MMO) and ammonia monooxygenase (AMO) enzymes, methanotrophs are capable of NH_4_^+^ oxidation, potentially producing NO_2_^−^ [79]. However, the maximum specific NO_2_^−^ production rate of methanotrophs is more than 20 times lower than the lowest NH_4_^+^ oxidizers’ production rates. Conversely, NH_4_^+^ oxidizers can also oxidize CH_4_, with the highest specific CH_4_ oxidation rates being more than five times lower than the lowest rates observed for aerobic methanotrophs. Furthermore, under hypoxic conditions, some Gammaproteobacterial methanotrophs can display reduction of NO_2_^−^ and NO_3_^−^ [80,81]. The nitrogen conversion capabilities of methanotrophs have been associated with N_2_O production [82], with N_2_O production increasing with increasing amounts of NO_2_^−^ and NO_3_^−^ under hypoxic conditions [81,82]. With the high relative abundances of methanotrophs, it is likely that some conversion of NH_4_^+^ to NO_2_^−^ and, subsequently, N_2_O via methanotroph MMOs occurred (further discussion in the Supplementary Materials).

In low-O_2_ reactors, CH_4_- and N-cycling organisms were represented on both membrane biofilms (Figure 4). Slightly higher O_2_ permeation rates could lead to better N removal by encouraging more partial nitritation and shifting more NH_4_^+^ to NO_2_^−^, likely the limiting substrate for anammox organisms in low-O_2_ reactors. Methanotroph–denitrifier synergy, i.e., methanotrophs’ production of intermediates (methanol, formaldehyde, and/or formate) and their use by denitrifiers, is associated with higher denitrification rates [83] and has been suggested to increase denitrification in wastewater-fed MBfRs [16,35,36,84]. Microbial community analyses in MBfRs capable of ME-SND indicate enrichment of aerobic methanotrophs of the genera *Methylomonas* and *Methylobacter*, which play key roles in CH_4_ conversion and the subsequent use of the resulting intermediates by denitrifiers [13,85]. *Methylomonas* and *Methylobacter* were among the most abundant methanotrophs identified in the high- and low-O_2_ reactors in this study.

**Figure 6:**
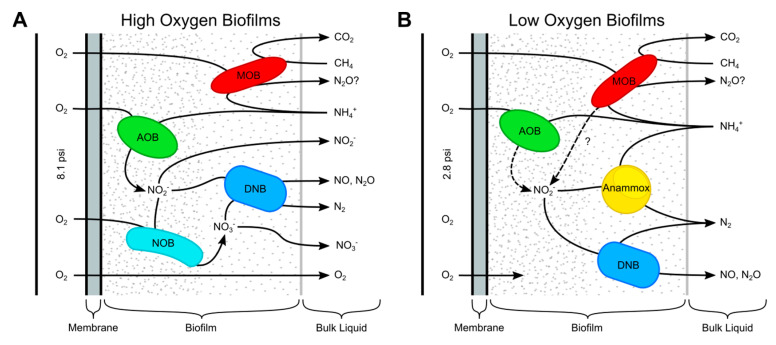
Simplified schematic of CH_4_- and N-cycling microbial groups in O_2_ membrane biofilms: (**A**) high-O_2_ biofilms and (**B**) low-O_2_ biofilms. Observed and potential N-cycle products shown. Membrane pressures are denoted in psi (gauge). Methane-oxidizing bacteria (MOB); ammonium-oxidizing bacteria (AOB); nitrite-oxidizing bacteria (NOB); putative denitrifying bacteria (DNB).

Anammox bacteria on O_2_ membrane biofilms suggest potential tolerance of higher O_2_ levels, which could be tested using O_2_ pressures between the 2.8 and 8.1 O_2_ psig pressures tested herein. In similar MBfR systems, the highest denitrification was observed at an O_2_ partial pressure of 5.5 psig, with optimal conditions for O_2_ depletion and sufficient methanotroph production of intermediates for denitrification activity [16]. Real-time measurement of bulk O_2_ or NH_4_^+^ to NO_2_^−^ levels and adjustment of membrane O_2_ pressures accordingly could optimize carbon and nitrogen removal [85]. Figure 6 shows a schematic of the CH_4_- and N-cycling microbial groups observed in the O_2_ membrane biofilms, along with potential CH_4_ and N species transformation.

## 5. Conclusions

This study investigated the removal of CH_4_ and N from NH_4_^+^- and CH_4_-rich AD effluent in single-stage continuous-flow aerobic MBfRs at two different O_2_ fluxes. Reactor performance and high-throughput 16S rRNA gene sequencing data showed that the O_2_ delivery rate affected N removal rates and shaped the biofilm microbial community. Low-O_2_ reactors had an enrichment of CH_4_- and N-cycling organisms on both membrane biofilms, and they had better N removal compared to high-O_2_ and control reactors. Complete nitrification and denitrification (e.g., SND) were observed in the low-O_2_ reactors, while partial nitrification, as shown by higher ratios of NO_2_^−^ to NO_3_^−^ levels, was observed high-O_2_ reactors. Slightly higher O_2_ permeation rates could lead to better N removal by encouraging more partial nitritation and shifting more NH_4_^+^ to NO_2_^−^, likely the limiting substrate for anammox organisms. The CH_4_ removal efficiencies were not dependent on initial methanotroph inoculation, and simultaneous removal of CH_4_ and nitrogen (ME-SND) from AD digestate liquids was demonstrated. Previous studies have typically relied on the use of synthetic wastewater as feed; here, we demonstrate an application of MBfRs using real wastewater capable of nitrogen removal, which shows promise for the application of membrane-based technologies. The use of MBfRs has been successfully applied at pilot scale for the effective removal of nitrogen from wastewater; however, further optimization is required to determine the ideal O_2_ operating conditions for partial nitritation–anammox to improve N removal efficiencies, minimize potential N_2_O emissions, and get closer to full-scale applications. Furthermore, for potential future implementation of MBfR treatment of nitrogen-rich wastewaters, there may be no need for the additional CH_4_ provided via the CH_4_ membrane, and pure O_2_ could be replaced by air for economic reasons.

## Figures and Tables

**Table 1:** Reactor and membrane conditions at startup.

Reactor Number	Reactor Condition	Methanotroph-Inoculated ^1^	O_2_ Membrane Pressure (psig) ^2^
R0	Control	−	N/A
R1, R2	High O_2_ (+)	+	8.1
R3, R4	High O_2_ (−)	−	
R5, R6	Low O_2_ (+)	+	2.8
R7, R8	Low O_2_ (−)	−	

^1^ All experimental conditions had duplicate reactors except for the control, with one reactor. Inoculated reactors indicated with a plus (+), while uninoculated reactors indicated with a minus (−). Reactor starting volumes consisted of 0.8 L of 1:1 diluted AD supernatant, diluted with DI water for the control, diluted with sterile NMS medium [39] and DI water for uninoculated experimental reactors, and diluted with methanotroph inoculate in fresh NMS medium and DI water (see Supplementary Materials and Table S4). ^2^ Pressures at startup shown; O_2_ pressures were constant throughout reactor operation. Initial CH_4_ membrane pressure was 11.6 psig and was changed starting in Period IV.

## Data Availability

The original data presented in this study are openly available in the National Center for Biotechnology Information (NCBI) database under submission: SUB12631397; BioProject ID: PRJNA928688.

## Supplementary Materials

The following supporting information can be downloaded at https://www.mdpi.com/article/10.3390/microorganisms12091841/s1: Supporting text, figures, and tables; Figure S1: Membrane bioreactor (A) COD, (B) HRT, (C) total suspended solids, (D) pH, and (E) PO_4_^3−^ throughout reactor operation; Figure S2: Concentrations of dissolved CH_4_, O_2_, and N_2_ in the AD feed tank; Figure S3: COD loading and removal rates of AD supernatant-fed membrane bioreactors; Figure S4: Reactor N_2_O production during Period III; Figure S5: Short-term NO_2_^−^ spike test conducted on day 180 (Period V); Figure S6: Membrane biofilm biomass (A and B) and thickness (C and D), day 206; Figure S7: Representative membrane biofilm cross-section images, control reactor; Figure S8: Representative membrane biofilm cross-section images, inoculated reactors; Figure S9: Representative membrane biofilm cross-section images, uninoculated reactors; Figure S10: Fisher diversity of microbial communities from AD supernatant and membrane bioreactors; Table S1: List of abbreviations and terms; Table S2: Membrane dimensions and characteristics; Table S3: Characteristics of secondary AD supernatant from IAWWTF; Table S4: Initial reactor conditions; Table S5: Reactor periods and operational changes; Table S6: Average solid retention time (SRT) of membrane bioreactors; Table S7: Nitrogen removal rates of membrane bioreactors for each operational period. References [20,38,39,86,87,88,89,90,91,92] are cited in the supplementary materials.
