# Supplementary material for: Treatment of Anaerobic Digester Liquids via Membrane Biofilm Reactors: Simultaneous Aerobic Methanotrophy and Nitrogen Removal
Source: Microorganisms. 2024 Sep 5;12(9):1841. doi: 10.3390/microorganisms12091841 (PMC11434332; doi:10.3390/microorganisms12091841)
Supplement: Supplementary file 1 [file microorganisms-12-01841-s001.zip › microorganisms-3156925-supplementary.pdf]

SUPPORTING INFORMATION FOR:

Treatment of Anaerobic Digester Liquids via Membrane Biofilm Reactors: Simultaneous Aerobic Methanotrophy and Nitrogen Removal

Egidio F. Tentori [1,2,*], Nan Wang [1], Caroline J. Devin [1] and Ruth E. Richardson [1]

[1] School of Civil and Environmental Engineering, Cornell University, Ithaca, NY 14853, USA; nw323@cornell.edu (N.W.); cjd245@cornell.edu (C.J.D.); rer26@cornell.edu (R.E.R.)
[2] Gradient, One Beacon St., Boston, MA 02108, USA
* Correspondence: eft35@cornell.edu

Number of Pages: 20

Number of Supporting Figures: 10

Number of Supporting Tables: 7

Table of Contents

Text

List of Figures

Figure S1. Membrane Bioreactor (A) COD, (B) HRT, (C) Total Suspended Solids, (D) pH and (E) PO_4^{3-} throughout Reactor Operation.

Figure S2. Concentration of Dissolved CH_4, O_2 and N_2 in AD Feed Tank.

Figure S3. COD Loading and Removal Rates of AD Supernatant-Fed Membrane Bioreactors.

Figure S4. Reactor N_2O Production During Period III.

Figure S5. Short-Term NO_2^- Spike Test Conducted on Day 180 (Period V).

Figure S6. Membrane Biofilm Biomass (A and B), Thickness (C and D), Day 206.

Figure S7. Representative Membrane-Biofilm Cross Section Images, Control Reactor.

Figure S8. Representative Membrane-Biofilm Cross Section Images, Inoculated Reactors.

Figure S9. Representative Membrane-Biofilm Cross Section Images, Uninoculated Reactors.

Figure S10. Fisher Diversity of Microbial Communities from AD Supernatant and Membrane Bioreactors.

List of Tables

Table S1. List of Abbreviations and Terms.

Table S2. Membrane Dimensions and Characteristics.

Table S3. Characteristics of Secondary AD Supernatant from IAWWTF.

Table S4. Initial Reactor Conditions.

Table S5. Reactor Periods and Operational Changes.

Table S6. Average Solid Retention Time (SRT) of Membrane Bioreactors.

Table S7. Nitrogen Removal Rates of Membrane Bioreactors for each Operational Period.

Secondary AD Supernatant

Secondary Anaerobic Digester (AD) supernatant was collected every 4-7 days directly from the secondary AD at the Ithaca Area Wastewater Treatment Facility (IAWWTF) in a gas-tight carboy, and stored in a walk-in refrigerator held at 4°C. Stored AD supernatant was used within 7 days of collection. Collected AD Supernatant was tested regularly throughout rector operation, Table S3 provides the number of samples collected for several parameters, mean, and standard deviation. The AD supernatant was characterized by having generally neutral pH, and high total suspended solids (TSS), chemical oxygen demand (COD), and ammonium levels, consistent with AD liquids. Fixed nitrogen compounds present in the AD supernatant were almost entirely ammonium; nitrite and nitrate were generally at the limit of detection or low relative to ammonium concentrations. Organic nitrogen content was measured three times and had an average of 256.90 mg n L^{-1}, about half the ammonium concentration, however organic nitrogen was not monitored regularly as it was beyond the scope of this study. Mean dissolved methane concentrations were close to saturation levels and dissolved oxygen concentrations were close to zero but not completely anaerobic, this was likely due to fugitive losses, and minor exposure to ambient air, which occurred during collection of the AD supernatant and sampling. Overall, the characteristics of the AD supernatant feed were generally consistent, with variability likely reflecting operational and process changes of the IAWWTF.

Methanotroph Inoculum

The mixed aerobic methanotroph culture used for inoculation consisted of a culture containing: *Methylosinus trichosporium* OB3b, *Methylocystis parvus* OBBP, *Methlyomicrobium album* BG8 and two *Methylomonas sp.*, grown under aerobic conditions in 1-L bottles with 600 mL of nitrate

mineral salts (NMS) medium [1] with CH_4 additions to bottle headspace as previously described [2]. The *M. trichosporium* OB3b, *M. parvus* OBBP, and *M. album* BG8 cultures were obtained from pure culture stocks. The *Methylomonas sp.* were isolated from incubations of Ithaca Area Wastewater Treatment Facility aeration tank wastewater and had 16S rRNA gene sequence similarities of 99% with *Methylomonas koyamae* LM6 and *Methylomonas* sp. ZR1. prior to startup.

Prior to startup, viability of the methanotroph inoculum culture was verified, samples were spun down, spent media was decanted and fresh NMS medium was added to achieve a concentration of 75 mg cell (dry weight) L^{-1}. All reactors started with 400 mL of AD supernatant, diluted 1:1 with a final volume of 800 mL. At startup, 200 mL of the resuspended culture in fresh NMS media and 200 mL of DI water was added to experimental reactors 1, 2, 5, and 6. The uninoculated experimental reactors (reactors 3, 4, 7, and 8) received 200 mL of fresh NMS media and 200 mL of DI water, while the control (reactor 0) only consisted of 1:1 diluted AD supernatant (Table S4).

Biomass and Biofilm Sampling

Suspended biomass samples from the control reactor (day 170) and AD feed influent (days 40 and 140) were pelleted, frozen and processed as previously described [2]. On day 206 membranes assemblies were removed and biofilm samples were collected from membrane segments using sterilized razor blades (Figure S6), biofilm thickness and mass were determined from triplicate membrane cross-section samples from images ($n > 32$ thickness measurements per membrane) using Inkscape (v 0.92.2) and ImageJ2 [3] and by vortexing membrane segments in 15-mL centrifuge tubes with 5 mL of milli-Q water and filtering using previously described methods [2]. Average reactor solid retention times (SRT) were determined from biofilm biomass measurements. Biofilm samples for nucleic acid extraction and microbial community analysis were collected in

2-mL tubes with 1 mL of DNase, RNase free water (Promega), vortexed, pelleted and stored at -80°C before use.

Due to the control reactor sloughing event, biofilm thickness measured at day 206 only represented ~35 days of growth and was not representative of the biofilm thickness throughout operation. Control reactor biofilm biomass was estimated from TSS samples collected before and on day 170 and the assumption a uniform membrane biomass distribution observed at day 206.

Reactor N_2O Discussion

Differences in N_2O production due to reactor inoculation were only observed for Low O_2 reactors, which were generally similar in overall reactor performance, biofilm thickness and microbial community. Under aerobic conditions, N_2O production in wastewater is attributed to denitrification NO_2^- by AOB and from hydroxylamine oxidation, and is favored at high NH_4^+ and low NO_2^- concentrations, and N_2O production increased in the presence nitrite and under low O_2 conditions [4]. N_2O emissions were lowest in conditions where heterotrophic denitrifiers had no oxygen inputs and no organic carbon limitation [4]. This could indicate that the differences in N_2O production could be due to differences in heterotrophic denitrifiers and incomplete denitrification in inoculated Low O_2 reactors. Given the high relative abundances of aerobic methanotrophs, differences in N_2O production could also be from NH_4^+ oxidation by aerobic methanotrophs, in addition to incomplete denitrification. O_2 is known to repress denitrification, inhibiting N-oxide reductase enzymes involved in denitrification [5]. AOB *Nitrosospira* sp. which have been correlated with N_2O emissions in soil was observed in High O_2, O_2-membrane biofilms with relative abundances ranging 0.31–10.81% [6]. Therefore, optimization of O_2 conditions is needed

for both maximizing total N-removal rates and minimizing N_2O emissions, as incomplete denitrification could lead to increased N_2O production.

Biofilm Thickness Discussion

The heterogeneity of membrane biofilm thickness (Figure S6) could due to local differences in reactor hydrodynamics as fluid shear stress (velocity) has been shown to proportionally increase biofilm densities [7]. Observed maximum MBfR biofilm thicknesses (~620–1925 μm) from literature were comparable to those observed in this study [8–10]. For aerated methanotrophic MBfRs, biofilm thicknesses <250 μm were fully penetrated by membrane and bulk liquid substrates, and at higher thicknesses, dual-substrate limitation conditions at the membrane-biofilm and biofilm-bulk liquid interfaces were observed [10]. In aerated MBfRs capable of SND, membrane-delivered O_2 penetrated 300–700 μm in biofilms with a 1600 μm thicknesses and AOB were found growing near membrane surface and not near the biofilm-bulk liquid interface (8). Membrane biofilm microbial stratification is dependent on both membrane pressures and bulk liquid substrate concentrations. Microbial biofilm stratification in this study was not determined, however based on biofilm characteristics and microbial composition it is likely the O_2-dependent microbial activity occurred near the membrane surface with the highest O_2 levels while bulk liquid O_2 allowed for methanotrophic activity on the CH_4 membrane.

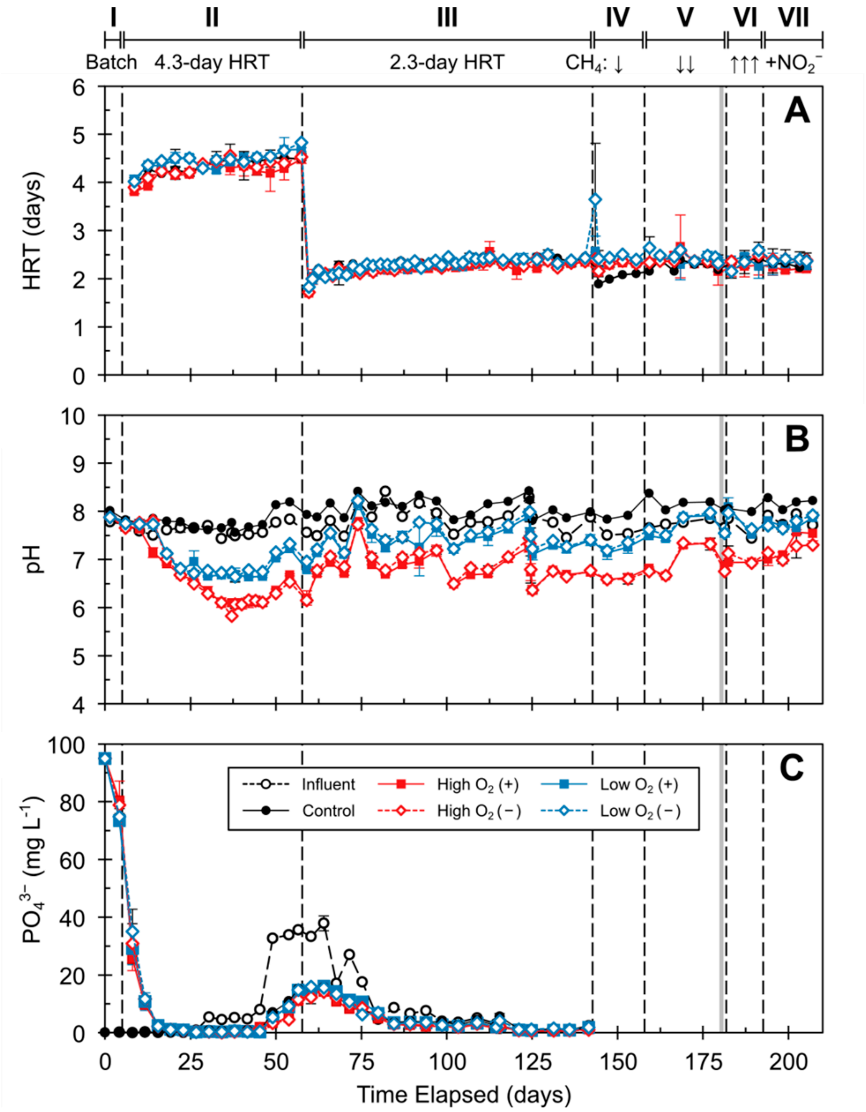

Figure S1. MBfR (A) HRT, (B) pH and (C) PO_4^{3-} throughout Operation. Vertical dashed black lines; operational periods; gray lines; batch activity periods; error bars, standard deviations from duplicate reactor measurements. Changes in operational period changes indicated above figure. PO_4^{3-} was only measured until day 152.

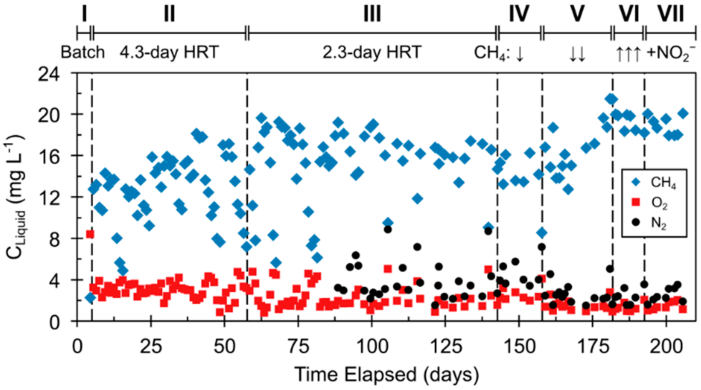

Figure S2. Concentration of Dissolved CH_4, O_2 and N_2 in the AD Feed Tank. Vertical dashed black lines: operational periods; activity period. Changes in operational period changes indicated above figure.

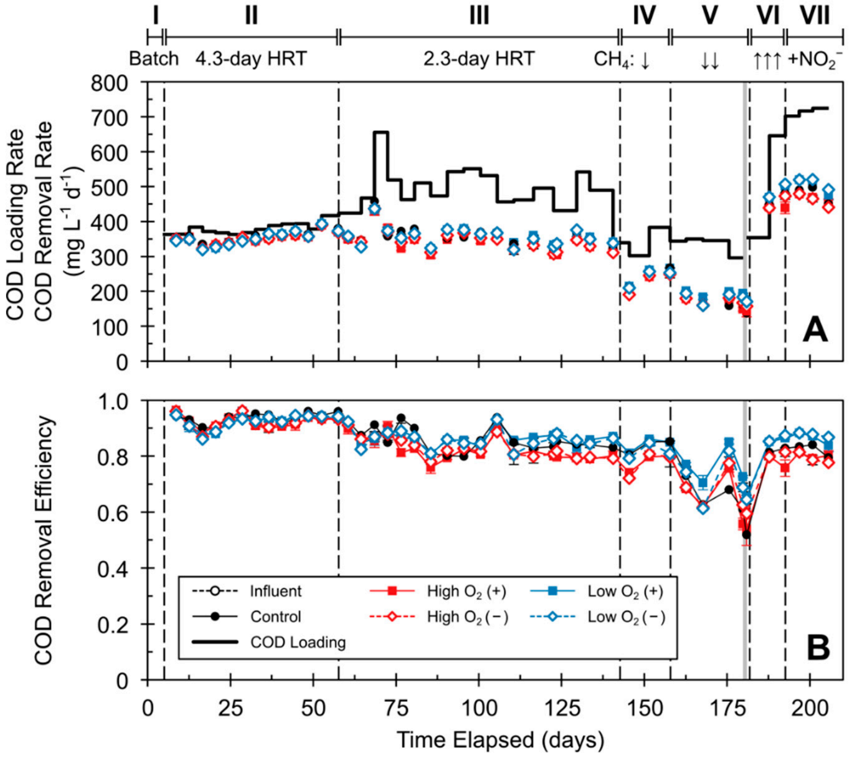

Figure S3. COD Loading and Removal Rates of MBfRs. (A) COD influent loading and removal rates; (B) COD Removal Efficiency. COD loading includes membrane CH_4 loading. Vertical dashed black lines; operational periods; gray lines; batch activity periods; error bars, standard

deviations from duplicate reactor measurements. Changes in operational period changes indicated above figure.

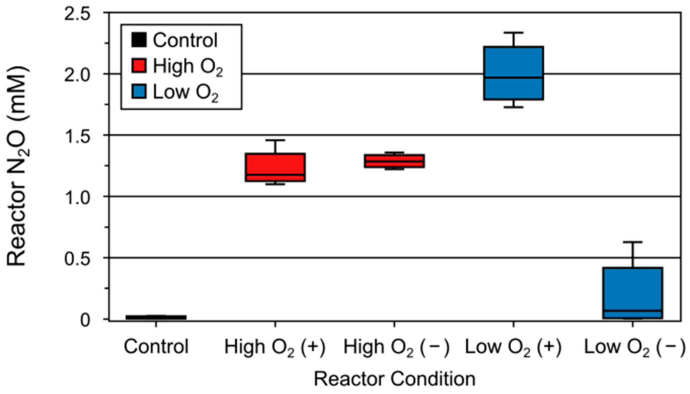

Figure S4. Reactor N_2O Production During Period III. Data determined from headspace samples collected on days 132, 135, and 138. Error bars represent 95% confidence intervals, median shown as a solid horizontal line.

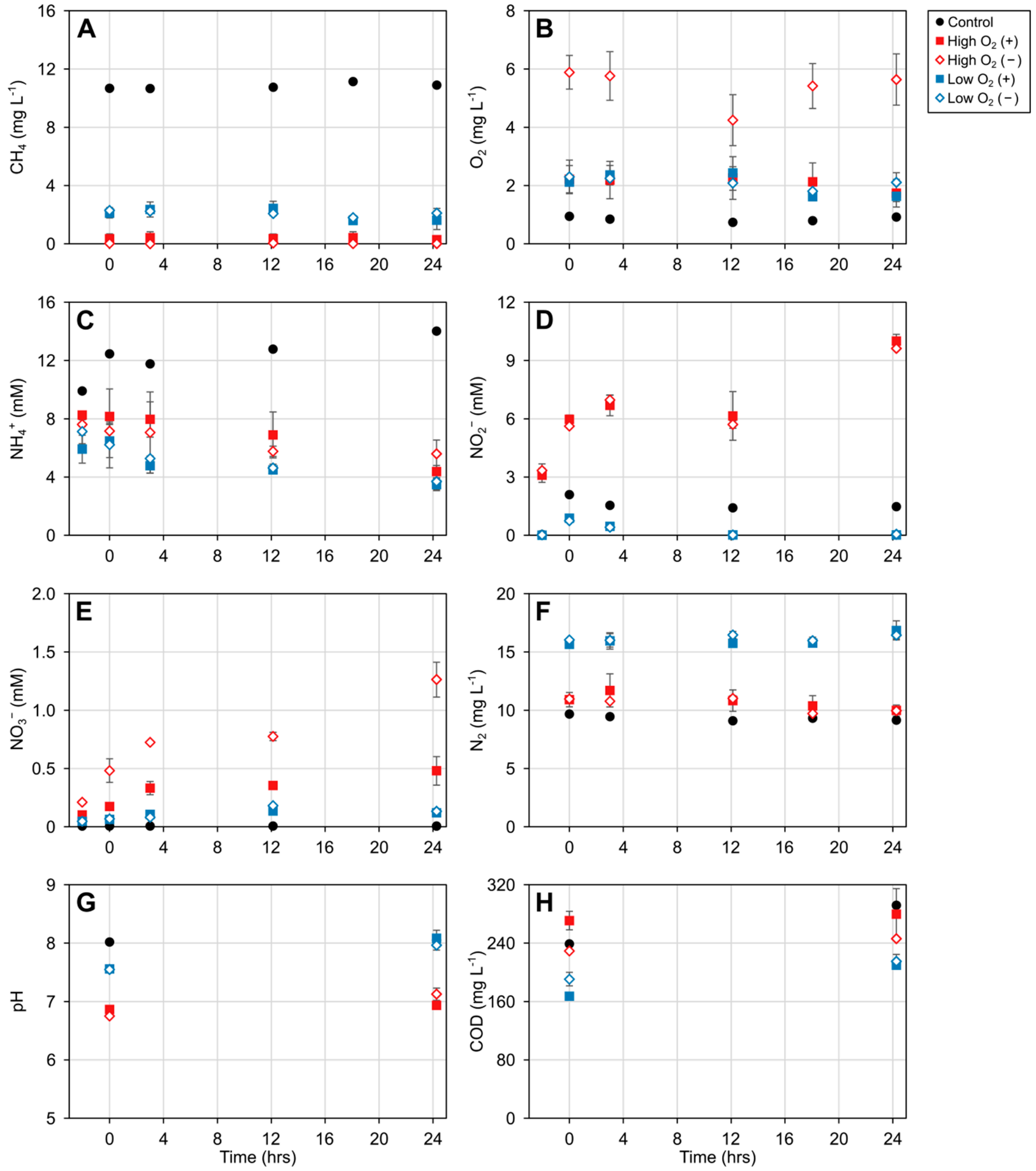

Figure S5. Short-Term NO_2^- Spike Test Conducted on Day 180 (Period V). NO_2^- was added at t = 0 to increase reactor concentrations by 2 mM. (A) Dissolved CH_4; (B) Dissolved O_2; (C) NH_4^+; (D) NO_2^-; (E) NO_3^-; (F) Dissolved N_2; (G) pH; (H) Chemical Oxygen Demand (COD). Error bars indicate standard deviation from reactor replicates.

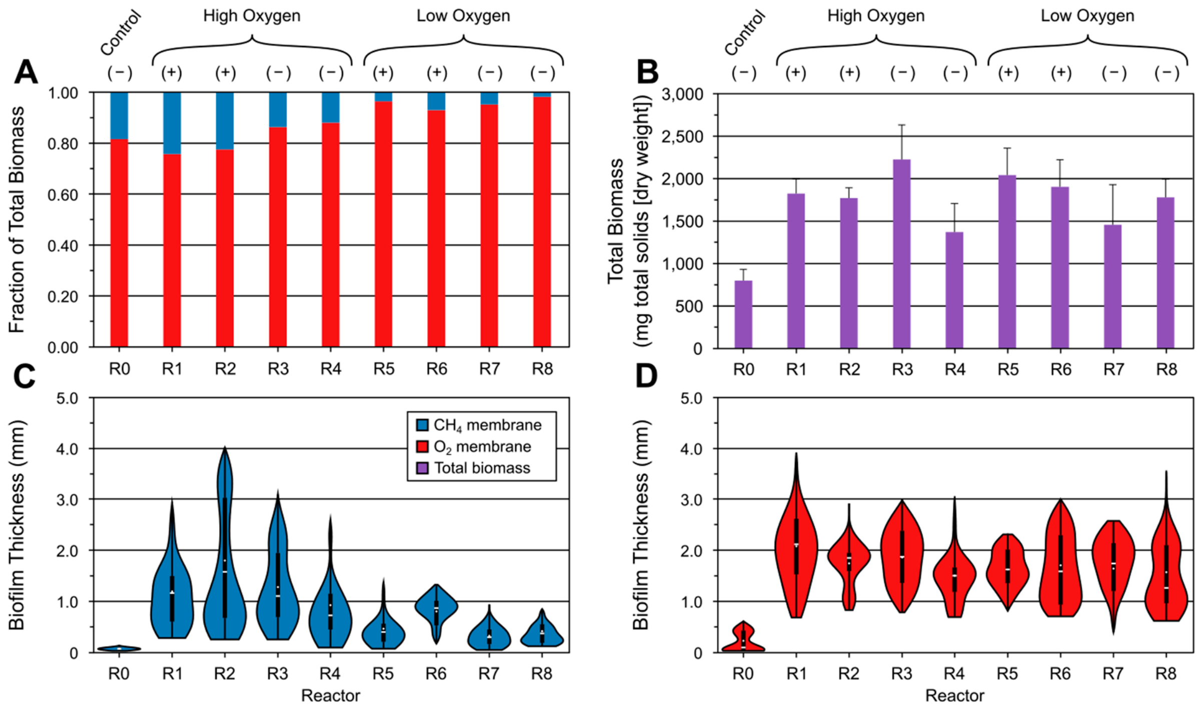

Figure S6. Membrane Biofilm Biomass (A and B), Thickness (C and D), Day 206. Control reactor (R0) biofilm thickness based on biofilm growth from day 170 to day 206. White points and lines in Biofilm Thickness (C and D) indicate mean and median values, respectively.

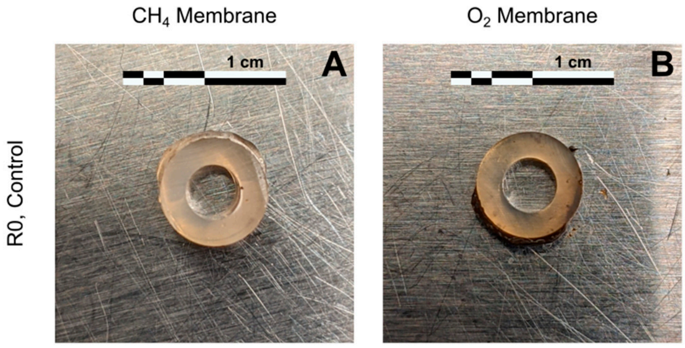

Figure S7. Representative Membrane-Biofilm Cross Section Images, Control Reactor. (A) R0 CH_4; (B) R0 O_2. Scale bar = 2 cm, 1 cm scale included.

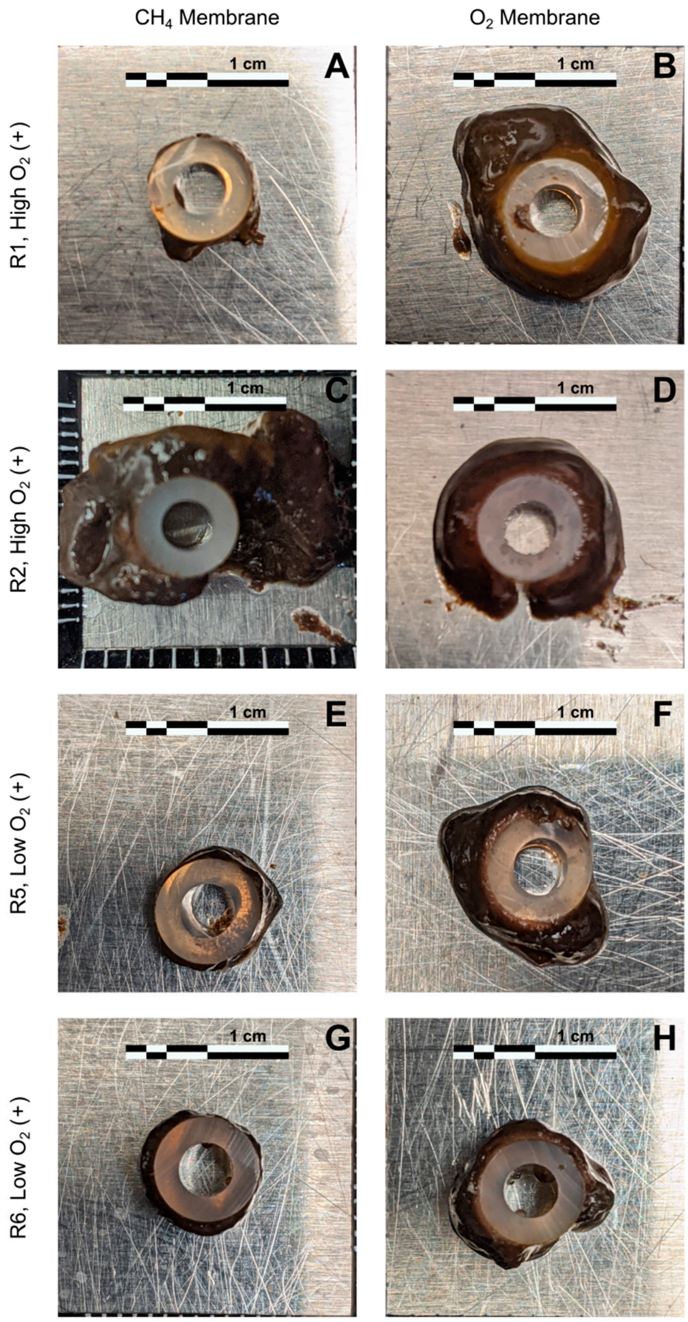

Figure S8. Representative Membrane-Biofilm Cross Section Images, Inoculated Reactors. (A) R1 CH_4; (B) R1 O_2; (C) R2 CH_4; (D) R2 O_2; (E) R5 CH_4; (F) R5 O_2; (G) R6 CH_4; (H) R6 O_2. Scale bar = 2 cm, 1 cm scale included.

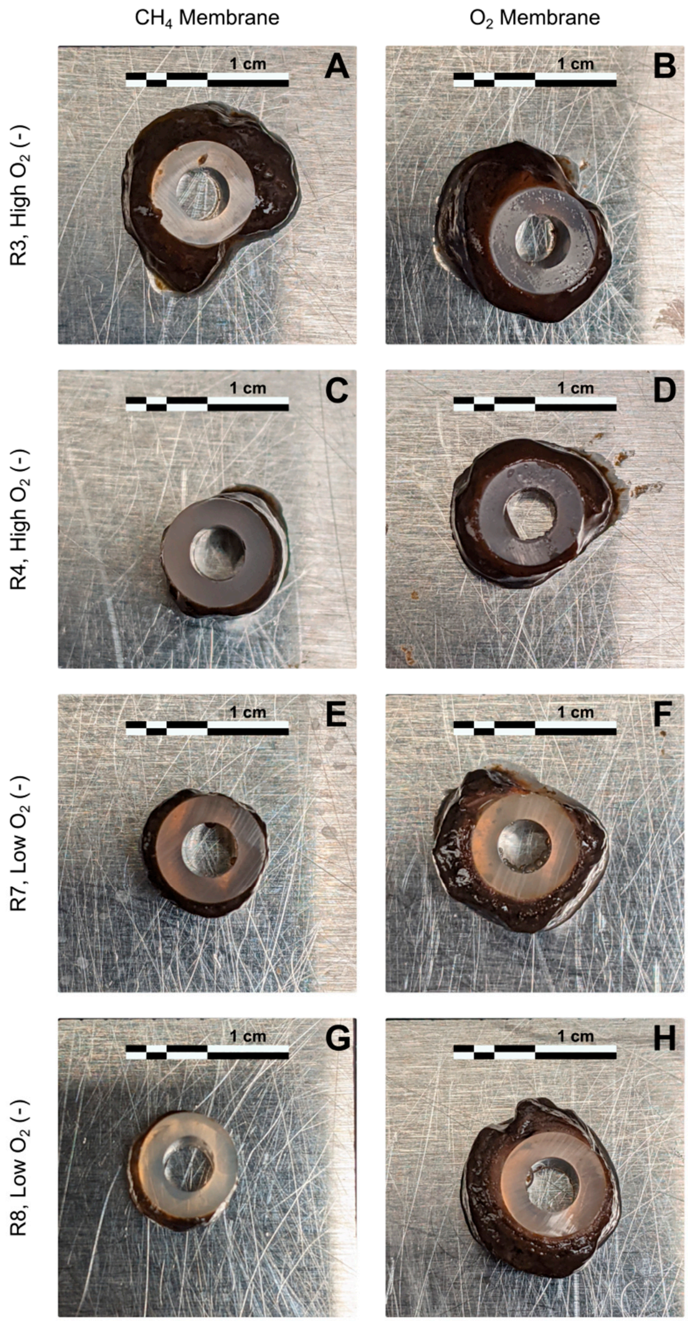

Figure S9. Representative Membrane-Biofilm Cross Section Images, Uninoculated Reactors. (A) R3 CH_4; (B) R3 O_2; (C) R4 CH_4; (D) R4 O_2; (E) R7 CH_4; (F) R7 O_2; (G) R8 CH_4; (H) R8 O_2. Scale bar = 2 cm, 1 cm scale included.

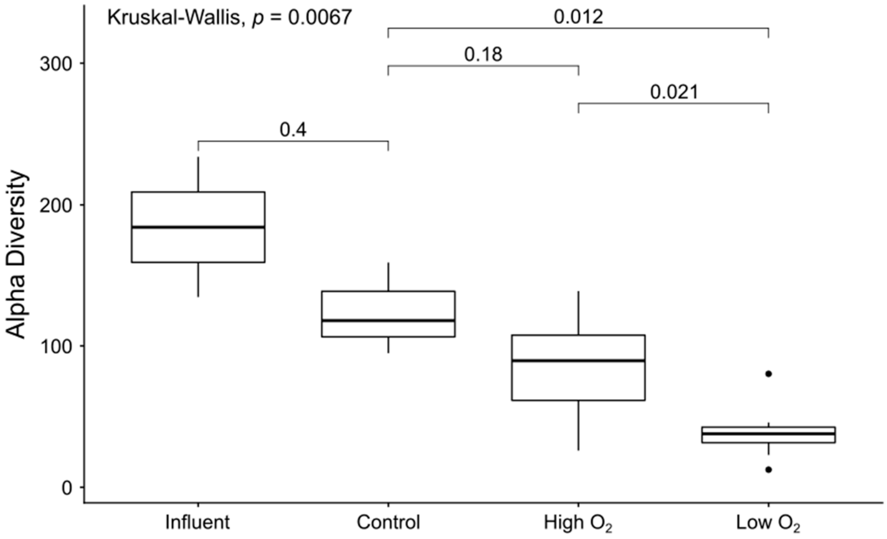

Figure 10. Fisher Diversity of Microbial Communities from AD Supernatant and Membrane Bioreactors.

Table S1. List of Abbreviations and Terms.

Abbreviation or Term	Description
AD	Anaerobic digestion
AD Digestate	Anaerobic digestion process residual liquids with high nitrogen content, including centrate and supernatant
AMO	Ammonia monooxygenase enzymes
AOB	Ammonium oxidizing bacteria
BRC	Cornell Biotechnology Resource Center
CH_4 and O_2 loading	Reactor methane and oxygen loading including membrane and influent loading
CH_4 and O_2 pressures	Reactor membrane methane and oxygen pressures throughout reactor operation
CH_4-cycling microbes	Microbes capable of transforming methane, part of the methane cycle
COD	Chemical Oxygen Demand
DAMO	Denitrifying anerobic methane oxidizers
DNB	Denitrifying bacteria
GHG	Greenhouse gas
High O_2	Experimental reactors which had O_2 Membrane Pressures of 8.1 psig
HRT	Hydraulic retention time
Influent loading rate	Rate of material entering the reactor in the influent.
Low O_2	Experimental reactors which had O_2 Membrane Pressures of 2.8 psig
MBfR	Membrane Biofilm Reactors (MBfRs)
ME-SND	Methane oxidation coupled with simultaneous nitrification and denitrification
MMO	Methane monooxygenase enzymes
MOB	Methane oxidizing bacteria
N_2-N	Nitrogen present as nitrogen gas

N-cycling microbes	Microbes capable of transforming nitrogen species, part of the nitrogen cycle
NH_4^+-N	Ammonia nitrogen, refers to the nitrogen present as ammonium
NO_2^--N	Nitrite nitrogen, refers to the nitrogen present as nitrite
NO_3^--N	Nitrate nitrogen, refers to the nitrogen present as nitrate
NOB	Nitrite oxidizing bacteria
N-Removal	Nitrogen removal,
N_{Tot}	Total Inorganic Nitrogen ($N_{Tot} = NH_4^+$-N + NO_2^--N + NO_3^--N)
PCoA	Principal Coordinate Analysis
psig	Gage pressure in pounds per square inch
Removal rate	Rate of material being removed or transformed in the reactor.
SND	Simultaneous nitrification and denitrification
SRT	Solid retention time
TSS	Total Suspended Solids
UASB	Upflow anaerobic sludge blanket

Table S2. Membrane Dimensions and Characteristics.

Membrane[a]	Length (cm)	Surface Area (cm^2)	Specific Surface Area (cm^2 cm^{-3})	Permeation Coefficient, K_p[b] (cm d^{-1})	Permeation Rate[c] (mg d^{-1})
CH_4	20	39.90	3.73	98.4	82.0
O_2	60	119.69	11.20	36.0	360.0
Total	80	159.59	14.94	-	-

[a]Membrane material consisted of platinum-cured, HelixMark® PharmaFocus™ PDMS tubing (3.18 mm ID × 6.35 mm OD × 1.59 mm wall thickness; Freudenberg Medical, Carpinteria, CA).
[b]Permeation coefficients obtained from Tentori *et al.* (1).
[c]Based on given K_p, tubing dimensions and a membrane pressure of 1 atm.

Table S3. Characteristics of Secondary AD Supernatant from IAWWTF.

Chemical Compound or Property	Number of Measurements	Mean[a]	Standard Deviation	Units
Total suspended solids	15	1131.73	300.93	mg L^{-1}
Chemical oxygen demand (COD)	45	633.36	222.87	mg L^{-1}
Ammonium (NH_4^+)	46	403.17	84.67	mg-N L^{-1}
Nitrite (NO_2^-)	38	0.08	0.05	mg-N L^{-1}
Nitrate (NO_3^-)	38	0.23	0.19	mg-N L^{-1}
Organic Nitrogen	3	9.27	11.99	mg-N L^{-1}
Phosphate (PO_4^{3-})	34	256.90	58.25	mg L^{-1}
Dissolved Oxygen	47	2.15	1.16	mg L^{-1}
Dissolved Methane	47	14.71	3.78	mg L^{-1}
pH	47	7.74	0.22	
Total alkalinity (as $CaCO_3$)	3	3325.00	606.71	mg L^{-1}

[a]Values determined from AD supernatant material collected throughout reactor operation.

Table S4. Initial Reactor Conditions.

Reactor Number	Reactor Condition	Initial Volume (mL)				Total Volume (mL)
		Methanotroph Inoculum	NMS Medium	DI Water	AD Supernatant	
R0	Control	-	-	400	400	800
R1, R2	High O_2 (+)	200	-	200	400	800
R3, R4	High O_2 (-)	-	200	200	400	800
R5, R6	Low O_2 (+)	200	-	200	400	800
R7, R8	Low O_2 (-)	-	200	200	400	800

Table S5. Reactor Periods and Operational Changes.

Period	Day	Time Elapsed (days)	CH_4 Pressure[a] (psi)	RT[a] (days)	Mol Ratios (CH_4/O_2)		Notes
					Low	High	
I	0–5	5.00	11.44	N/A			Initial batch
II	5–58	52.18	11.49	4.34	0.92	0.33	
III	58–143	84.97	11.65	2.29			
IV	143–158	15.20	7.88	2.29	0.63	0.22	
V	158–182	23.92	5.95	2.29	0.47	0.17	Day 180: batch test, NO_2^- added
VI	182–193	10.79	15.59	2.29			
VII	193–206	13.05	15.70	2.29	1.27	0.46	5 mM NO_2^- in feed

[a]Average CH_4 membrane pressures and retention times are provided for a given period.

Table S6. Average Solid Retention Time (SRT) of Membrane Bioreactors.

	Average SRT (days)				
Period	Control	High O_2 (+)	High O_2 (-)	Low O_2 (+)	Low O_2 (-)
I	N/A	N/A	N/A	N/A	N/A
II	62.4 ± 7.5	74.9 ± 2.0	78.0 ± 13.0	73.1 ± 5.1	62.6 ± 5.0
III – VII	12.1 ± 4.0	31.7 ± 4.1	34.1 ± 9.3	56.8 ± 19.2	53.4 ± 2.7

SRT determined from biofilm biomass data, total suspended solids data and hydraulic retention times, averages for corresponding periods shown.

Table S7. Nitrogen Removal Rates of Membrane Bioreactors for each Operational Period.

		Nitrogen Removal Rate (mg-N L^{-1} day^{-1})				
Period		Control	High O_2 (+)	High O_2 (-)	Low O_2 (+)	Low O_2 (-)
I		-	-	-	-	-
II	*median*	8.43	11.48	11.77	13.50	14.91
	mean	15.50	14.27	13.74	18.27	18.52
	max	30.34	26.87	30.96	36.22	35.98
III	*median*	16.84	21.43	24.66	25.92	25.13
	mean	19.00	22.98	25.36	27.51	29.44
	max	39.77	56.46	56.83	62.39	66.00
IV	*median*	7.89	26.12	24.68	31.60	30.17
	mean	8.12	27.54	24.61	30.39	29.64
	max	9.85	37.99	34.60	34.64	33.68
V	*median*	15.40	26.52	14.44	45.76	37.36
	mean	14.99	25.19	20.45	51.65	47.03
	max	32.52	49.47	42.58	93.79	93.05
VI	*median*	29.57	26.33	41.34	50.66	44.02
	mean	29.57	27.71	41.90	50.92	45.79
	max	38.70	34.90	48.74	68.79	56.06
VII	*median*	30.74	22.76	29.94	82.56	82.13
	mean	25.81	25.67	32.23	80.55	81.97
	max	36.63	35.09	48.43	91.73	88.43

REFERENCES

1. Whittenbury, R.; Phillips, K.; Wilkinson J. 1970. Enrichment, Isolation and Some Properties of Methane-utilizing Bacteria. J Gen Microbiol 61:205–218. DOI: 10.1099/00221287-61-2-205.
2. Tentori, E.F.; Richardson, R.E. 2020. Methane Monooxygenase Gene Transcripts as Quantitative Biomarkers of Methanotrophic Activity in *Methylosinus trichosporium* OB3b. Appl Environ Microbiol 86. DOI: 10.1128/AEM.01048-20.
3. Rueden, C.T.; Schindelin, J.; Hiner, M.C.; DeZonia, B.E.; Walter, A,E,; Arena, E.T.; Eliceiri, K.W. 2017. ImageJ2: ImageJ for the next generation of scientific image data. BMC Bioinformatics 18:529. DOI: 10.1186/s12859-017-1934-z.
4. Wunderlin, P.; Mohn. J.; Joss, A.; Emmenegger, L.; Siegrist, H. 2012. Mechanisms of N_2O production in biological wastewater treatment under nitrifying and denitrifying conditions. Water Res 46:1027–1037. DOI: 10.1016/j.watres.2011.11.080.
5. Zumft, W.G. 1997. Cell biology and molecular basis of denitrification. Microbiol Mol Biol Rev MMBR 61:533–616. DOI: 10.1128/.61.4.533-616.1997.
6. Lourenço, K.S.; Cassman, N.A.; Pijl, A.S.; van Veen, J.A.; Cantarella, H.; Kuramae, E.E. 2018. *Nitrosospira* sp. govern nitrous oxide emissions in a tropical soil amended with residues of bioenergy crop. Front Microbiol 9:674. DOI: 10.3389/fmicb.2018.00674.
7. Pereira, M.O.; Kuehn, M.; Wuertz, S.; Neu, T.; Melo, L.F. 2002. Effect of flow regime on the architecture of a *Pseudomonas fluorescens* biofilm. Biotechnol Bioeng 78:164–171. DOI: 10.1002/bit.10189
8. Terada, A.; Hibiya, K.; Nagai, J.; Tsuneda, S.; Hirata, A. 2003. Nitrogen removal characteristics and biofilm analysis of a membrane-aerated biofilm reactor applicable to high-strength nitrogenous wastewater treatment. J Biosci Bioeng 95:170–178. DOI: 10.1016/s1389-1723(03)80124-x.
9. dos Santos, L.M.F.; Livingston, A.G. 1995. Membrane-attached biofilms for VOC wastewater treatment I: Novel *in situ* biofilm thickness measurement technique. Biotechnol Bioeng 47:82–89. DOI: 10.1002/bit.260470110.
10. Rishell, S.; Casey, E.; Glennon, B.; Hamer, G. 2004. Characteristics of a methanotrophic culture in a membrane-aerated biofilm reactor. Biotechnol Prog 20:1082–1090. DOI: 10.1021/bp049902k.
